# Retroperitoneal Urinoma Spontaneously Drained in the Scrotum Repaired with Gracilis Muscle Flap: A Case Report

**DOI:** 10.1155/2012/597839

**Published:** 2012-12-01

**Authors:** Emanuela Altobelli, Alfredo Maria Bove, Federico Sergi, Maurizio Buscarini

**Affiliations:** Urology Department, Università Campus Biomedico di Roma, Via Alvaro del Portillo 200, 00128 Rome, Italy

## Abstract

*Aim.* To report a unique case of retroperitoneal urinoma extending to the scrotum through the spermatic cord and successfully treated with nephrostomy, drainage, and gracilis muscle flap.

## 1. Case Report

A 66-year-old male patient was referred to us from the medical oncology department for a scrotal wound with purulent discharge. 

**Figure 1:**
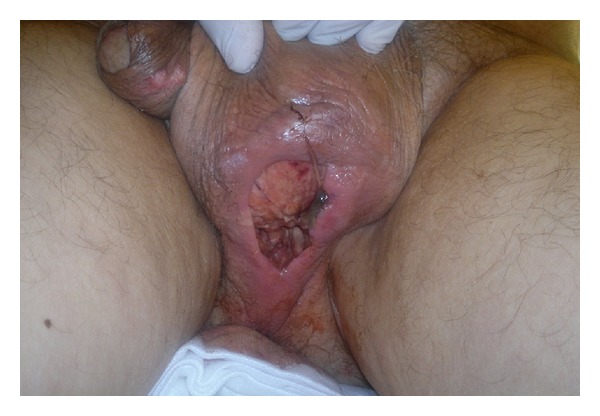
Perineal wound.

On physical examination, he presented with severe asthenia, abdominal pain, cushingoid aspect, tender abdomen mostly on lower quadrants, and reddened, swollen scrotum with foul smelling 4 × 5 cm defect. The wound presented pus and haematic discharge and exposed spermatic cord and testis (Figure 1). After culture samples were taken, the wound was debrided, irrigated with saline solution, and packed with gauzes.

**Figure 2:**
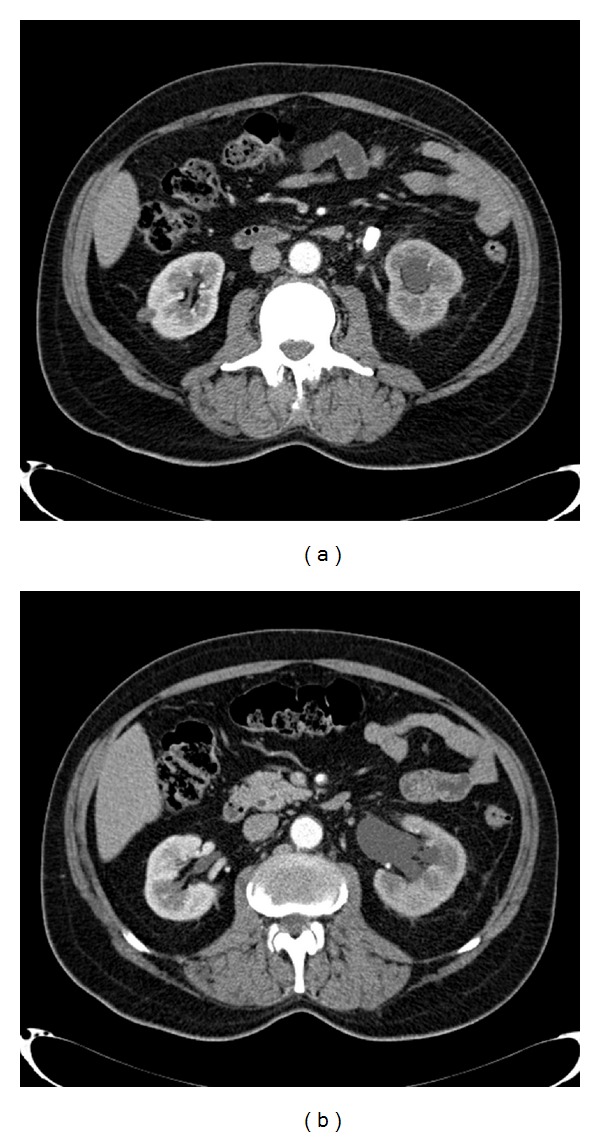
(a) Abdomen CT scan: left ureteral stone. (b) Abdomen CT scan: left hydronephrosis.

Patient's urologic history had started two months earlier, when during oncological followup for metastatic prostate cancer, a CT scan showed a 15 mm left ureteral stone causing hydronephrosis (Figures 2(a) and 2(b)). He was scheduled for left laser ureteral lithotripsy and double J stent positioning. The first procedure was only partially successful, and a second-look procedure was scheduled 4 weeks later. 

Before the latter, he started external beam radiotherapeutic treatment for D11-L1 bone metastases, and after 9 Gy in 3 fractions, he developed the scrotal abscess. His therapy included daily oral intake of 8 mg of Dexamethasone, infusion of 66 mg of Taxoter twice a month, and monthly infusion of 4 mg of Zoledronic acid. Blood gas showed normal pH and hyperglycemia confirmed by laboratory (793 mg/dL). Blood tests showed also hyponatremia (127 mEq/L), normal renal function (creatinine 0.81 mg/dL), Hb 10.9 g/dL, Hct 34.3%, WBCs 7.730/uL, PLTs 173.000/uL, and a normal coagulation pattern. Peripheral venous access and transurethral catheter were inserted. Insulin drip, intravenous rehydration with 2000 cc of Normo Saline, and empiric antibiotic therapy with Clindamycin 600 mg and Ceftazidime 1 g every 8 hours were administrated. 

**Figure 3:**
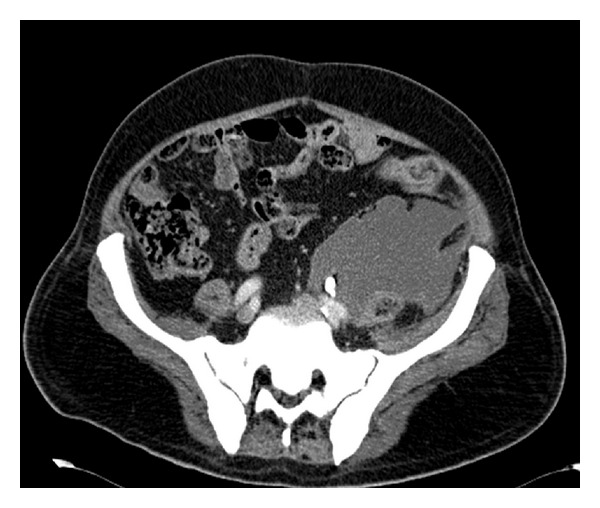
Abdomen CT scan: left urinoma, left ureteral stone, and left double J stent.

Abdominal CT-scan showed (Figure 3) in the distal part of left ureter a 13 mm stone with a well-positioned double J stent and a 12 cm urinoma anterior to iliopsoas muscle as for ureteral leakage according to increased attenuation of urinoma in delayed imaging. The urinoma extended from the ileopsoas muscle through spermatic funicle and drained in the scrotum. 

Left percutaneous nephrostomy and abdominal drain were immediately positioned. Culture samples were taken.

**Figure 4:**
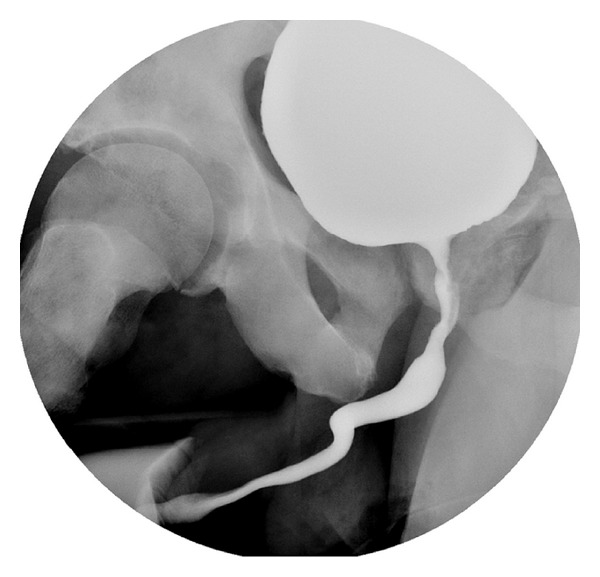
Voiding cystourethrogram.

A voiding cystourethrogram (VCUG) was performed this showed no evidence of urine leakage (Figure 4).

Wound culture samples showed bacterial growth of *E. coli* sensible to Ceftazidime and the abdominal sample obtained by the drain a *Staphylococcus epidermidis* growth sensitive to Erythromycin. Antimicrobial therapy was modified, Clindamycin was stopped, and Erythromycin 600 mg was orally administrated every 8 hours. 

**Figure 5:**
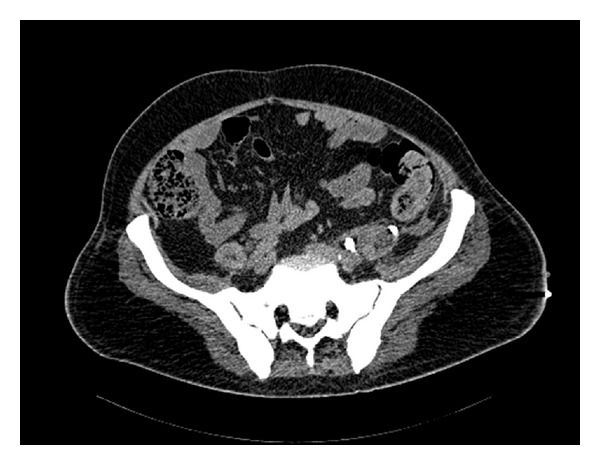
Abdominal CT scan: absence of the urinoma.

A second CT scan was performed 10 days after nephrostomy and drain insertion showing complete absence of the urinoma (Figure 5). Urine culture was negative for bacterial growth.

Patient was scheduled for wound debridement and closure with gracilis muscle perineal flap.

## 2. Surgical Technique

**Figure 6:**
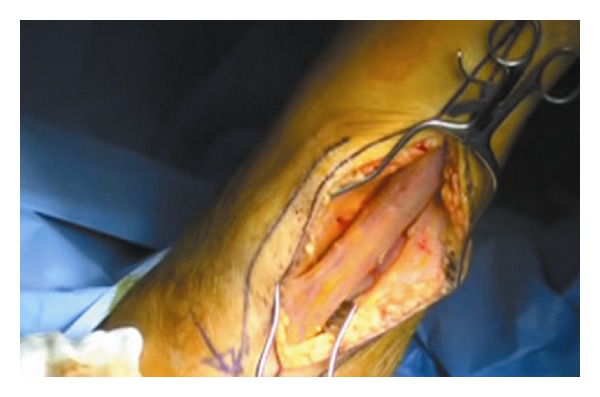
Gracilis muscle.

**Figure 7:**
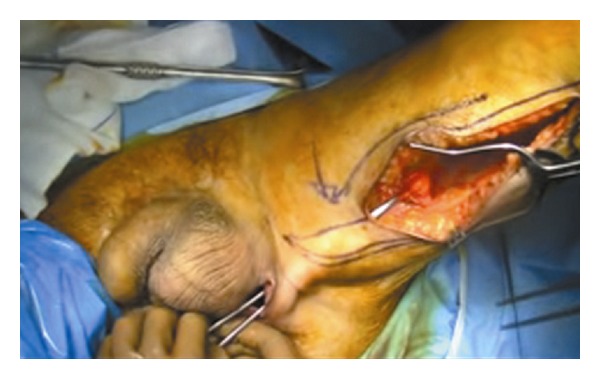
Gracilis flap tunneled.

The patient was placed in lithotomy position, and after sterile draping, cystoscopy and proctoscopy were performed without evidence of any fistulae. A longitudinal incision was made along the medial side of the thigh, making it possible to identify the gracilis muscle (Figure 6) and to mobilize it according to the McCraw technique [1]. The width of the muscle flap was approximately 3 cm and the length 10 cm. To reach the perineal area, the muscle flap was tunneled (Figure 7) and fixed into position with 3-0 Polyglactin suture without tension. After fixation, the thigh incision was closed in one layer over a drain, with minimal subcutaneous undermining, provided by adduction of the thigh. Same procedure was performed for the perineal wound.

## 3. Results

**Figure 8:**
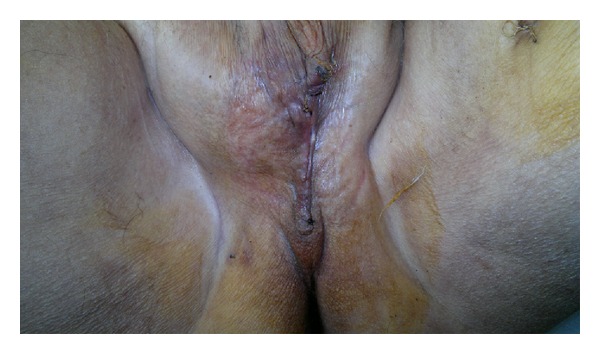
Perineal scar.

The duration of the procedure was 2 hours. Drains were removed on day 3 postoperatively. Patient began ambulation on postoperative day 3 and was able to seat 1 week after the surgery. The flap survived with good wound healing. The hospitalization after the procedure was 5 days. Time to complete healing of the donor site and perineal defect (Figure 8) was 10 and 15 days, respectively. The patient was able to move well with no limitation of motion. 

After negative previous transnephrostomic antegrade pyelography, performed 1 month after surgery, transurethral catheter and left nephrostomy were removed. 

During the 7 months of followup, the patient was satisfied with the functional and aesthetic outcome.

## 4. Discussion

Self-limiting urinoma formation due to spontaneous extravasation is rare. McCraw et al. described subcapsular renal urinoma in a patient with gynaecological malignancy treated with previous radiotherapy [1]. Anderson et al. described a case of spontaneous urinoma caused by a ureteral calculus [2]. Our case is the first in the literature describing a case of retroperitoneal urinoma in a patient with ureteral calculus and previous radiotherapy, with spontaneous drain through scrotum. A mininvasive approach was used to successfully treat the urinoma. A surgical approach was instead needed to repair the scrotal wound especially is not a very commonly used English word. When the testes are exposed, scrotal reconstruction is required not only for cosmetic reasons but also for functional and psychological reasons [3]. Functional preservation is one of the cornerstones of scrotal reconstruction. Spermatogenesis and hormonal production of Leydig cells can be maintained only by covering exposed testicles [4]. The gracilis muscle flap is well known to provide the best reconstructive option for major scrotal defects [5]. The gracilis muscle is a broad sheet-type shape that can be wrapped around the defect and does not add much bulk to the reconstructed scrotum. The coverage of the scrotal defect site can protect testicles without increasing the temperature. As a muscle flap, it has an infection-resistant nature and represents a useful choice for reconstruction in the contaminated perineum, as in our patient. It is a relatively simple and reliable procedure. 

## 5. Conclusions

A mininvasive approach is prefered in the treatment of urinoma; a surgical approach is needed in case of scrotal wound.
